# Supplementary material for: Evaluation of Fecal Coliform Prevalence and Physicochemical Indicators in the Effluent from a Wastewater Treatment Plant in the North-West Province, South Africa
Source: Int J Environ Res Public Health. 2020 Sep 2;17(17):6381. doi: 10.3390/ijerph17176381 (PMC7503972; doi:10.3390/ijerph17176381)
Supplement: Supplementary file 1 [file ijerph-17-06381-s001.pdf]

Table S1. Wastewater treatment plant (WWTP) removal efficiency with standard deviations.

Months	*E. coli* (count/100 mL)					Ammonia (mg/L)					COD (mg/L)					Nitrate (mg/L)					Ortho-phosphate (mg/L)				
	Secondary Effluent	* Std. dev.	**F.E	*Std. dev.	***Red %	Raw Inlet	*Std. dev.	**F.E	*Std. dev.	***Red %	Raw Inlet	*Std. dev.	**F.E	*Std. dev.	***Red %	Raw Inlet	*Std. dev.	**F.E	*Std. dev.	***Red %	Raw Inlet	*Std. dev.	**F.E	*Std. dev.	***Red %
May-19	4.93×10^4	1.01×10^5	70	84.32	99.86	43.64	4.86	0.25	0.29	99.43	674	111.7	27.06	15.62	95.99	9.24	3.05	7.66	2.36	17.1	5.47	0.84	0.49	0.47	91.04
Jun-19	2.06×10^4	3.48×10^4	46	50.74	99.78	39.81	11.82	0.57	0.6	98.57	694	242.22	31.6	13.95	95.45	8.29	3.22	8.52	2.41	-2.77	4.97	1.03	0.61	0.57	87.73
Jul-19	1.03×10^4	8.22×10^3	36	25.58	99.65	41.24	9.27	0.2	0.18	99.52	709	180.08	27.42	10.96	96.13	10.6	3.48	11.17	1.93	-5.38	5.62	1.43	0.38	0.22	93.24
Aug-19	1.39×10^4	1.27×10^4	35	39.16	99.74	51.98	14.14	0.22	0.52	99.58	727	198.59	28.52	14.53	96.08	7.93	2.49	9.75	2.21	-22.95	6.12	1.37	0.36	0.18	94.12
Sep-19	7.45×10^3	7.47×10^4	68	133.41	99.09	39.21	18.07	0.09	0.16	99.77	792	307.66	33.03	8.91	95.83	8.65	4.49	8.94	1.49	-3.35	6.92	2.32	0.48	0.21	93.06
Oct-19	1.17×10^5	9.72×10^4	54	89.1	99.95	40.46	7.82	0.99	1.32	97.55	805	195.87	27.77	6.76	96.55	11.82	5.92	4.71	4.71	60.15	7.13	1	0.35	0.26	95.09
Nov-19	5.42×10^5	7.15×10^5	103	439.15	99.98	49.58	27.43	7.76	4.9	84.35	741	341.78	27.27	11	96.32	10.19	4.16	1.19	0.91	88.32	7.31	1.6	0.22	0.21	96.99
Dec-19	3.07×10^5	3.74×10^5	98	431.72	99.97	27.64	13.75	5.56	6.22	79.88	413	309.75	24.23	7.23	94.13	6.19	3.85	1.97	0.91	68.17	4.27	2.08	0.32	0.72	92.51
Jan-20	5.67×10^4	6.19×10^4	123	450	99.78	28.06	15.21	0.89	1	96.83	206	126.22	15.32	8.58	92.56	3.35	1.46	2.28	0.75	31.94	3.36	1.19	0.45	1.53	86.61
Feb-20	2.29×10^4	9.57×10^4	76	201.78	99.67	45.04	15.18	2.91	1.63	93.54	356	184.46	17.17	7.37	95.18	4.75	1.57	1.66	0.79	65.05	4.3	0.89	0.51	0.56	88.14
Mar-20	2.42×10^5	2.85×10^5	51	184.58	99.98	31.75	14.13	3.97	3.35	87.5	292.64	171.71	20.81	8.64	92.89	5.23	2.96	2.6	0.91	50.29	3.69	1.46	0.46	0.41	87.53
****Ave	1.26×10^5	2.95×10^5	69	249.09	99.77	39.86	3.63	2.13	3.63	94.23	582.69	11.86	25.47	11.86	95.19	7.84	3.96	5.5	16.1	31.51	5.38	0.62	0.42	0.62	91.46

** F.E = Final Effluent; *** Redtn % = Reduction percentage; **** Ave = Average
